# Osteoblast Cell Response to Naturally Derived Calcium Phosphate-Based Materials

**DOI:** 10.3390/ma11071097

**Published:** 2018-06-27

**Authors:** Valentina Mitran, Raluca Ion, Florin Miculescu, Madalina Georgiana Necula, Aura-Catalina Mocanu, George E. Stan, Iulian Vasile Antoniac, Anisoara Cimpean

**Affiliations:** 1 Department of Biochemistry and Molecular Biology, University of Bucharest, 91-95 Spl. Independentei, 050095 Bucharest, Romania; valentinamitran@yahoo.com (V.M.); rciubar@yahoo.com (R.I.); necula.madalina92@gmail.com (M.G.N.); 2 Department of Metallic Materials Science, Physical Metallurgy, University Politehnica of Bucharest, 313 Splaiul Independentei, J Building, District 6, 060042 Bucharest, Romania; mcn_aura@hotmail.com (A.-C.M.); antoniac.iulian@gmail.com (I.V.A.); 3 S.C. Nuclear NDT Research & Services S.R.L, Department of Research, Development and Innovation, 104 Berceni Str., Central Laboratory Building, District 4, 041912 Bucharest, Romania; 4 National Institute of Materials Physics, Laboratory of Multifunctional Materials and Structures, Atomistilor Str., No. 405A P.O. Box MG 7, 077125 Măgurele-Bucharest, Romania; george_stan1@yahoo.com

**Keywords:** osteoblast, biocompatibility, naturally derived calcium phosphate, seashell, dolomitic marble

## Abstract

The demand of calcium phosphate bioceramics for biomedical applications is constantly increasing. Efficient and cost-effective production can be achieved using naturally derived materials. In this work, calcium phosphate powders, obtained from dolomitic marble and *Mytilus galloprovincialis* seashells by a previously reported and improved Rathje method were used to fabricate microporous pellets through cold isostatic pressing followed by sintering at 1200 °C. The interaction of the developed materials with MC3T3-E1 pre-osteoblasts was explored in terms of cell adhesion, morphology, viability, proliferation, and differentiation to evaluate their potential for bone regeneration. Results showed appropriate cell adhesion and high viability without distinguishable differences in the morphological features. Likewise, the pre-osteoblast proliferation overtime on both naturally derived calcium phosphate materials showed a statistically significant increase comparable to that of commercial hydroxyapatite, used as reference material. Furthermore, evaluation of the intracellular alkaline phosphatase activity and collagen synthesis and deposition, used as markers of the osteogenic ability of these bioceramics, revealed that all samples promoted pre-osteoblast differentiation. However, a seashell-derived ceramic demonstrated a higher efficacy in inducing cell differentiation, almost equivalent to that of the commercial hydroxyapatite. Therefore, data obtained demonstrate that this naturally sourced calcium-phosphate material holds promise for applications in bone tissue regeneration.

## 1. Introduction

The understanding of processes involved in bone biomineralization has led lately to the development of improved biomimetic synthesis methods and the production of a new generation of biomaterials. Calcium carbonate (CaCO_3_) emerged as a sustainable bioresource for various calcium phosphates synthesis along with coralline hydroxyapatite [1] and soon became a target subject for the extensive research in the reconstructive orthopedics field. Now both the marine (corals, seashells, cockleshells, cuttlefish bones) and terrestrial forms (marble, land snails) of CaCO_3_ are known as an eco-friendly, sustainable and geographically available resource, but improvements are still necessary in terms of calcium phosphates synthesis parameters [2]. The use of natural materials and structures for medical purposes was motivated by the limitations of synthetic materials generation in regard to the necessary mechanical features and integrity [3,4,5,6]. In terms of targeted precursors, dolomitic marble and *Mytilus galloprovincialis* seashells, autochthonously available, represent two different calcium carbonate polymorphic forms—calcite and aragonite—which have similar chemical composition, except for the Mg ion content that defines the dolomitic marble species [2]. Research carried out has shown that both marine and terrestrial resources can be an appropriate resource for biological phase-pure and thermally stable CPC (calcium phosphate ceramics) production [7]. 

Given that the mineral component of the human bone is apatite, calcium phosphate materials have been readily preferred because of their role in the bone remodeling process. In terms of the chemical composition, calcium phosphates currently used as biomaterials are classified as: calcium hydroxyapatite (HA), α and β tricalcium phosphate (α- and β-TCP), biphasic calcium phosphates, represented by mixtures of HA and β-TCP, and unsintered or calcium-deficient apatite [8].

The use of CPC provides advantages in terms of new bone formation processes, which are influenced by crystallinity and the Ca/P mole ratio, strongly related to the release of calcium and phosphate ions required for bone mineralization. In addition, extensive literature has shown that calcium phosphate bioceramic pellets promote both osteogenesis and osteointegration, in correlation to the samples’ chemistry and surface load, as well as topography [9,10,11]. However, it should be noted that the target application of these ceramics is to transiently replace natural bone [9].

The growth of osteoblasts on such surfaces requires certain dimensions and an interconnected pore structure. Complementary to the physical structure, chemical composition affects the performance of HA ceramics, so that large crystal decks and high CaO content decrease biocompatibility [12]. At the same time, HA obtained from natural sources is non-stoichiometric and may incorporate other ions, for example CO_3_^2−^, traces of Fe^2+^, Na ^+^, Mg^2+^, F^−^, and Cl^−^, so making them more similar to natural apatite compared to pure stoichiometric HA, and therefore more bioactive [13]. Hence, the presence of these ions influences numerous biochemical reactions related to bone metabolism. For instance, the presence of Na^+^ and Mg^2+^ is very important for bone development, their absence leading to bone loss and fragility [14]. Possible disadvantages of these materials relate to the differences regarding Ca/P mole ratio, particle size, morphology, phase composition, thermal stability or trace elements composition when different sources are used or due to batch variation [15]. 

HA is a calcium phosphate ceramic with a similar crystallographic structure to the naturally sourced apatite and has proved successful when involved in surgical and dental bone defects restoration for the last 20 years [16,17]. Besides, HA exhibits excellent biological and chemical affinity for bone tissue, and animal studies have shown that this ceramic is in the long-term biocompatible without harmful side effects, immunogenicity or inflammatory responses [18,19,20,21,22,23,24]. From a thermodynamic point of view, HA is the most stable calcium phosphate ceramic [25]. However, the costs associated with the synthesis of HA from inorganic Ca- and P-based sources are frequently high. Hence, an increasing number of articles have been devoted to the synthesis, characterization, and application of naturally derived HA, which has demonstrated superior biological behavior when compared to chemically synthesized HA. 

In this context, the aim of this study was to evaluate the biological properties of calcium phosphate-based materials derived from dolomitic marble and *Mytilus galloprovincialis* seashell in comparison with those of commercial hydroxyapatite. The in vitro response of MC3T3-E1 pre-osteoblasts to the developed naturally derived materials revealed their ability to support cell adhesion and viability, maturation of the actin cytoskeleton, as well as cell proliferation and differentiation. 

## 2. Materials and Methods

### 2.1. Ceramic Synthesis

The tested samples for this research were synthesized through a parametrically improved Rathje method [26]. The experimental setup involved the following stages: (i) thermal dissociation of autochthonous seashells (*Mytilus galloprovincialis*, CaCO_3_) and dolomitic marble (CaMg(CO_3_)_2_) resulting in calcium oxide (CaO) powder was carried out; (ii) after hydration of the CaO, the resultant calcium hydroxide (Ca(OH)_2_) powder (10 g) was mixed with 200 mL distilled water and a stoichiometrically calculated amount of phosphoric acid (5.5 mL), was added dropwise at room temperature; (iii) magnetic stirring of the obtained slurry was conducted for 2 h and (iv) aging and drying of the powder was carried out at room temperature and in an electric oven (200 °C), respectively [3,27]. The resultant ceramic powders were ground in a planetary mill with an agate bowl and balls and sorted using standardized granulometric sieves (<100 μm particle size). They were further subjected to cold isostatic pressing with a force of 10 MPa in a Φ30 mm mold. Pellets were then kept at room temperature for 24 h and sintered at 1200 °C for 10 h. Subsequently, samples were ground using abrasive paper P2500 to obtain parallel planar surfaces. Some of them were exposed to the three-point bending test as a method for fractographic surfaces achievement.

### 2.2. Characterization of the Synthesized Products

For both the synthesis and sintering stages, products were investigated through:

− Morpho-compositional analysis: by Scanning Electron Microscopy—Philips Xl 30 ESEM TMP (FEI/Phillips, Hillsboro, OR, USA) coupled with Energy Dispersive Spectroscopy—EDAX Sapphire spectrometer. An acceleration voltage of 25 kV and working distance of 10 mm were used. The SEM and EDS investigations were conducted on three randomly chosen areas.
− Structural analyses: by (1) X ray diffraction, XRD—Bruker D8 (Bruker Corporation, Billerica, MA, USA). An Advance diffractometer was used equipped with a LynxEye detector, in Bragg-Brentano geometry, with Cu K_α_ (λ = 1.5418 Å) radiation with the scattered intensity being scanned in the 2θ range of 10–60°, with a step size of 0.04° and a dwell time of 1 s; and (2) Fourier Transform Infrared Spectroscopy: FT-IR were acquired on a Perkin Elmer Spectrum BX II spectrometer (PerkinElmer, Inc., Waltham, MA, USA), in attenuated total reflectance (ATR) mode (PikeMiracle head); the spectra being recorded over the range 4000–500 cm^–1^, at a resolution of 4 cm^−1^ and using 32 scans/experiment.

As a comparison measure, we chose a commercial hydroxyapatite (Merck KGaA, Darmstadt, Germany) as a reference material. Preliminary results had already been published [3,27,28,29,30], but were confirmed by the new investigations carried out in the present study.

After thermal treatment, wettability was evaluated by contact angle (CA) measurements with 3 different wetting agents: water, diiodomethane (DIM) and ethylene glycol (EG), using a Krüss Drop Shape Analyser—DSA100 (A. Krüss Optronic GmbH, Hamburg, Germany). The experiments were conducted at constant parameters of 20 ± 1 °C temperature and room humidity of 45 ± 5% with images being captured at 1 s after wetting agent droplet deposition. The results comprised an average of 3 determinations/sample. 

### 2.3. Cell Culture Experiments

Mouse pre-osteoblasts (MC3T3-E1, ATCC^®^, CRL-2593^TM^) were directly seeded on the top of the ceramic samples (marble- and seashell-derived calcium phosphate-based materials, and commercial HA) at a density of 1 × 10^4^ cells·cm^−2^ excepting the osteogenic differentiation studies where an initial cell density of 4 × 10^4^ cells·cm^−2^ was used. Prior to cell seeding, the tested samples (smaller discs with a diameter of 10 mm and an area of 0.785 cm^2^) were sterilized at 180 °C for 1 h, washed four times for 15 min with Dulbecco’s Modified Eagle’s Medium (DMEM, Sigma-Aldrich Co., St. Louis, MO, USA) and then placed in 12-well plates. After that, the cells attached to the substrates were incubated in DMEM supplemented with 10% fetal bovine serum (Gibco (Life Technologies Corporation, Grand Island, NY, USA)) and 1% (*v*/*v*) penicillin/streptomycin (10,000 units mL^−1^ penicillin and 10 mg mL^−1^ streptomycin) (Sigma Aldrich) in a humidified atmosphere of 5% CO_2_ at 37 °C for specific time points. Pre-osteoblast differentiation was assayed both in standard culture medium and osteogenic medium containing ascorbic acid (AA, 50 μg/mL) and β-glycerophosphate (β-GP, 5 mM). All experiments were performed in triplicate.

#### 2.3.1. Assessment of the Cellular Survival and Proliferation

Possible cytotoxic effects of the analyzed samples were evaluated by quantification of lactate dehydrogenase (LDH) released in the culture medium by the cells grown in contact with their surfaces. This test was performed after 1 and 4 days of culture, by using a “LDH-based In Vitro Toxicology Assay Kit” (Sigma-Aldrich Co. St. Louis, MO, USA), according to the manufacturer’s protocol. Absorbance was recorded at 490 nm using a microplate reader (Thermo Scientific Appliskan, Vantaa, Finland), low OD values indicating the materials’ capacity to sustain cellular survival. 

Cell viability/ proliferation was quantified at the same time points by means of MTT (3-(4,5-Dimethyl-2-thiazolyl)-2,5-diphenyl-2H-tetrazolium bromide) assay. At specified time points the cells were incubated with 1 mg mL^−1^ MTT solution for 3 h at 37 °C. Then, the MTT solution was removed and the insoluble purple formazan produced by metabolically active viable cells was solubilized with dimethyl sulfoxide and the absorbance of dye measured at 550 nm using a microplate reader (Thermo Scientic Appliskan, Vantaa, Finland).

The above quantitative assays were accompanied by a qualitative assay consisting of cell staining with a LIVE/DEAD Cell Viability/Cytotoxicity Assay Kit (Molecular Probes, Eugene, OR, USA). This assay was performed in accordance with the manufacturer’s instructions. Briefly, after 1 and 4 days of culture, the analyzed samples were incubated in a solution containing 2 mM calcein-AM and 4 mM ethidium homodimer-1 (EthD-1) for 10 min at room temperature, in the dark. Afterwards, the samples were washed with phosphate buffered saline (PBS, Gibco) and examined under an inverted microscope Olympus IX71 (Olympus, Tokyo, Japan) to detect viable (green fluorescence) and dead (red fluorescence) cells. The fluorescent images were captured using a Cell F Image acquiring system.

#### 2.3.2. Evaluation of MC3T3-E1 Cell Adhesion and Morphology

The adhesion and morphological appearance of MC3T3-E1 cells grown on each ceramic sample were assessed by immunoreactive staining of vinculin and labeling of actin filaments with phalloidin coupled with Alexa Fluor 546, at 3 h and 24 h post-seeding. In this assay, the pre-osteoblasts grown on the substrates were fixed with 4% paraformaldehyde, permeabilized and blocked with 0.1% Triton X-100/2% Bovine Serum Albumin (BSA) in PBS and incubated with mouse anti-vinculin monoclonal antibody, dilution 1:50 (Santa Cruz Biotechnology, Dallas, TX, USA) in PBS containing 1.2% BSA. After washes with PBS, they were further incubated with Alexa Fluor 488-conjugated goat anti-mouse IgG antibody (Invitrogen, Eugene, OR, USA) in PBS containing 1.2% BSA for 1 h. After this step, phalloidin conjugated with Alexa Fluor 546, 20 µg/mL (Invitrogen, Eugene, OR, USA) was added for actin staining. Also, the nuclei were marked with 2 µg/mL 6-diamidino-2-phenylindole (DAPI, Sigma-Aldrich Co., Steinheim, Germany). Labeled samples were washed with PBS and examined under an inverted microscope equipped with epifluorescence (Olympus IX71, Olympus, Tokyo, Japan). Representative, microscopic images were captured using the Cell F software.

#### 2.3.3. Measurement of the Pre-Osteoblast Differentiation

To determine whether the three synthesized ceramics supported differentiation of MC3T3-E1 cells in osteoblasts, ALP activity and collagen synthesis were measured under both standard and osteogenic culture conditions. The intracellular ALP activity was determined after 7 and 14 days of culture by using Alkaline Phosphatase Activity Colorimetric Assay Kit (BioVision, Milpitas, CA, USA), as we reported in a recent paper [31]. Briefly, 80 µL from cellular lysate were mixed with 50 µL of 5 mM p-nitrophenylphosphate (pNPP) and incubated for 1 h at room temperature in the dark. After this step, 20 µL of stop solution was added to each sample and the absorbance was measured at 405 nm using a microplate reader (Thermo Scientific Appliskan, Vantaa, Finland). A standard curve was used to determine the concentrations of the reaction product. ALP activity was calculated using the formula: ALP activity (U/mL) = A/V/T, where A represents the amount of p-nitrophenol (pNP) expressed by the samples (in mol), V is the volume of cell lysate used in reaction (in mL) and T is the reaction time (in min). Also, the protein concentrations were measured for each sample using the Bradford reaction and ALP activity was normalized to 1 µg of protein.

The measurement of collagen synthesis and deposition on the cell supporting biomaterials was performed at 3-weeks post-seeding by staining with Sirius Red, as previously described [32]. Briefly, samples were washed with PBS and fixed with 10% paraformaldehyde. After three washes with deionized water, samples were maintained in 0.1% solution of Sirius Red (Bio-Optica, Milano, Italy) for 1 h at room temperature and washed again. Next, samples were dried for 24 h in air. Finally, the stain was dissolved in 0.2 M NaOH/methanol (1:1) and the optical density was recorded at 540 nm. 

### 2.4. Statistical Analysis

Statistical analysis of data was performed with GraphPad Prism software (Version 3.03, GraphPad, San Diego, CA, USA) using one-way ANOVA with Bonferroni’s multiple comparison tests. All values are expressed as means ± standard deviation (SD) of three independent experiments and differences at *p* < 0.05 were considered statistically significant. 

## 3. Results and Discussion

### 3.1. Materials’ Characterization

**Figure 1:**
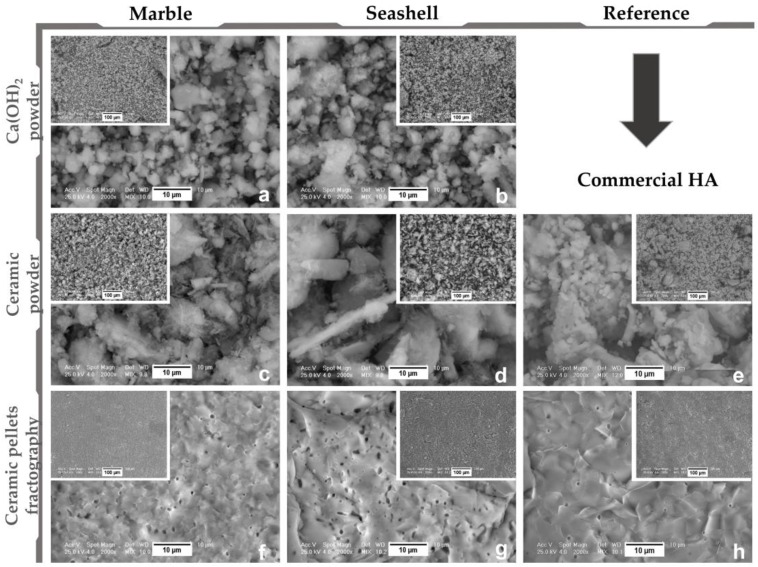
Representative micrographs for marble, seashell and reference material derived products: Ca(OH)_2_ (**a**,**b**), Ceramic powder (**c**–**e**), Ceramic pellets fractographic surface (**f**–**h**).

After thermal dissociation and hydration stages, chemical analysis proved the presence of only calcium hydroxide characteristic elements and the conservation of Mg composition for marble-derived samples. Microstructure analysis of calcium hydroxide powder (Figure 1a,b) revealed the tendency of fine particles to agglomerate as irregularly sized and shaped crystals (1–10 μm). Compared to the regular and fine microstructure of Merck HA, marble and seashell derived bioceramics (Figure 1c–e) displayed mainly clustered grains and few dispersed polyhedral and needle-like ones with variable sizes in the range of 1–20 μm. The sintering process induced an irregular micrometric layered morphology with a high compaction degree and accentuated porosity (~1 μm min. pore diameter), similar to the reference samples. Supplementary, fractography analysis indicated a brittle intercrystalline fracture of both sample types (Figure 1f–h) with prominent and defined grain boundaries. In addition, it revealed a porous structure, with augmented internal microporosity (0.5–1 μm pore diameter), far more accentuated for seashell derived samples. After thermal treatment pellet shrinkage of linear dimensions was estimated at ~15% (samples diameter of ~26.5 mm and thickness of 3 mm).

**Figure 2:**
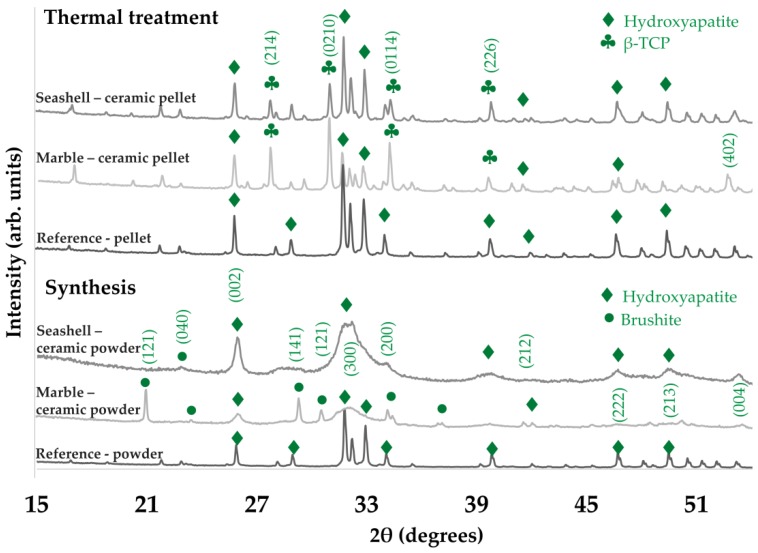
Representative XRD patterns for seashell, marble and reference material derived products.

**Figure 3:**
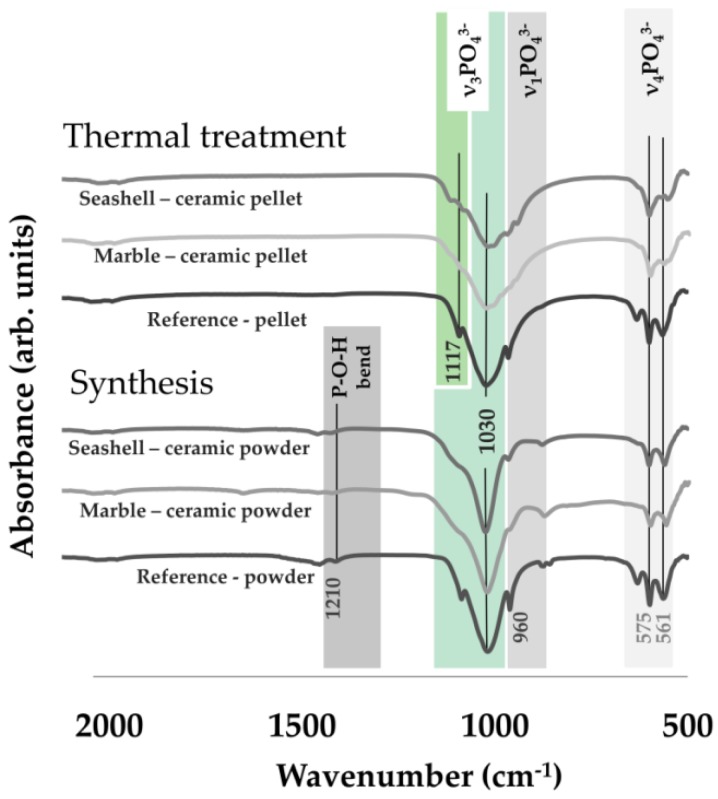
Representative FT-IR spectra for seashell, marble and reference material derived products.

Post synthesis, EDS results (see Table 1) exposed a typical calcium phosphate composition and values ranging from 1.50–1.53 for the Ca/P mole ratio. This correlated with the XRD patterns revealing a biphasic composition (HA and brushite) only for marble-derived samples, which was expected given the inhibitory effect of Mg on HA precipitation. More to the point, FT-IR spectra confirmed these results as for all samples, HA characteristic bands were detected and in particular, more intense peaks corresponding to hydrogen phosphate ions were attributed to brushite presence. Further thermal treatment improved and modulated the chemical and structural composition. As a key indicator for derived bioceramics performance, the Ca/P mole ratio increased from 1.60–1.62, which is close to the reference value of HA (1.67), with no alteration of the elemental composition. In terms of structure, given the high temperature treatment, both XRD and FT-IR analyses (Figure 2 and Figure 3) showed a biphasic transformation for all synthesized materials: i.e. with HA coexisting with β-TCP and with no trace of residual, cytotoxic compounds [3]. 

Taken together, the results summarize the potential of the adapted Rathje method for naturally derived calcium phosphates synthesis, even from marble precursors, and the importance of thermal treatment for modulated, enhanced morpho-compositional, and structural features.

**Figure 4:**
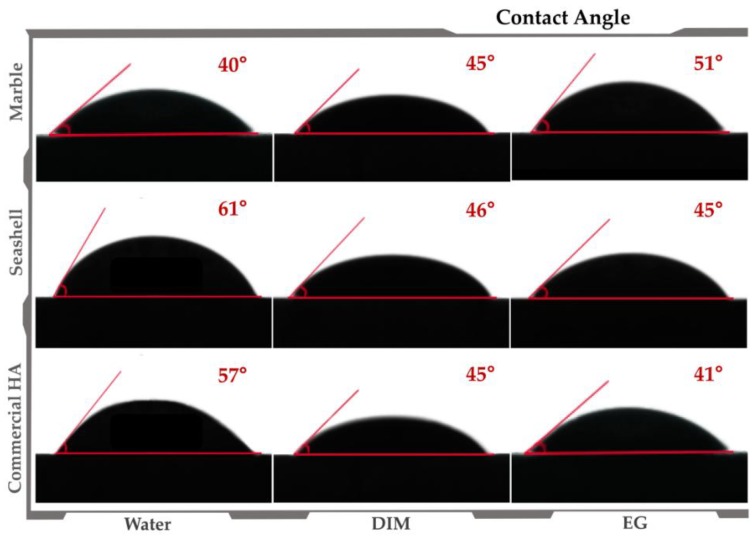
Contact angle assessment for marble- and seashell- derived ceramics and commercial HA samples.

Regarding the wettability of the sintered pellets, contact angle (CA) values varied from 40–51° and 45–61° for marble and seashell derived samples, respectively, and 41–57° for reference material (Figure 4). Therefore, results revealed a hydrophilic character (CA values < 90°) independent of the wetting agent and natural precursor, similar to the reference sample. However, given that surface morphology is a key factor for CA [33], the lowest values correspond to samples with an accentuated surface porosity (see Figure 1) and better surface wetting properties. Moreover, the ascending or descending trendline of CA found for marble and seashell derived samples is strongly related to the microporosity range of the samples, the surface preferential behavior to the wetting agent and the samples’ anisotropy.

### 3.2. Pre-Osteoblast Cell Response to Developed Ceramics

Cellular-based studies designed to evaluate the behavior of bone-derived primary cells or cell lines and mesenchymal stem cells (MSCs) represent a starting point for determining the biocompatibility of a material. In this study, we examined the viability/proliferation, adhesion and morphology, and differentiation of MC3T3-E1 pre-osteoblasts. These cells have been previously shown to exhibit stage specific genes as seen in vivo [34] and an osteoblast specific phenotype in contact with HA [35].

#### 3.2.1. Cellular Survival and Proliferation

The capacity of the developed naturally derived calcium phosphate-based materials to support the viability and proliferation of MC3T3-E1 pre-osteoblasts plated on their surfaces has been assessed by quantifying the amount of LDH released into the culture medium, MTT reduction levels and by distinct labeling of living cells with calcein AM and of dead cells with EthD-1. Commercial HA was used as a reference material in our experiments since previous studies showed that this material belonging to the calcium phosphate family exhibited excellent biocompatibility [36,37], osteoconductivity [37,38,39], and osteointegration [38] abilities. Furthermore, animal and clinical studies demonstrated its direct incorporation into bone [40] and physicochemical bonding without intervening connective tissue [37].

**Figure 5:**
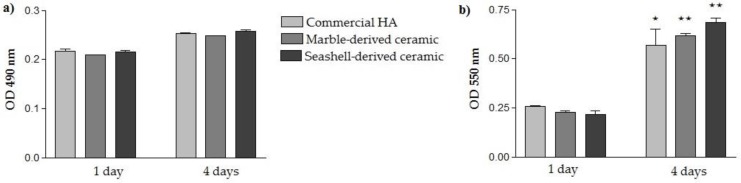
Cellular survival and proliferation of MC3T3-E1 pre-osteoblasts grown on tested substrates for 1- and 4-day culture periods. (**a**) LDH quantification as a measure of the ceramics’ cytotoxicity (*n* = 3, mean ± SD); (**b**) MTT results showing the proliferation of cells cultured on 0.785 cm^2^ of commercial HA, marble- and seashell-derived HA (*n* = 3, mean ± SD). ⋆ *p* < 0.05 for commercial HA, 4 day-culture vs. 1 day-culture, ⋆⋆ *p* < 0.01 for marble-derived ceramic, 4 day-culture vs. 1 day-culture, ⋆⋆ *p* < 0.01 for seashell-derived ceramic, 4 day-culture vs. 1 day-culture.

In a first set of experiments, the possible cytotoxicity effects exerted by marble- and seashell-derived materials in comparison to commercial HA were evaluated by LDH assay after 1- and 4-days of culture. LDH is a cytosolic enzyme that can be rapidly released into the cell culture medium upon damage of the plasma membrane resulting in an increase in the OD value. As noted in Figure 5a, low absorbance values were displayed by the cells grown in contact with all three analyzed ceramics suggesting that none of them exerted cytotoxic effects. Moreover, no significant differences between the samples were observed at both incubation times indicating that they equally sustain cellular survival. In addition, the results of the MTT assay (Figure 5b) demonstrate an upward trend in OD values from 1 day to 4 days post-seeding without any significant difference between cellular substrates. Therefore, the synthesized calcium phosphate-based materials elicited an increased proliferation potential of MC3T3-E1 pre-osteoblasts. This increase appeared more obvious for marble- and seashell-derived ceramics (*p* < 0.01) than for the reference material (*p* < 0.05) although no significant differences between the three analyzed materials were noticed. 

**Figure 6:**
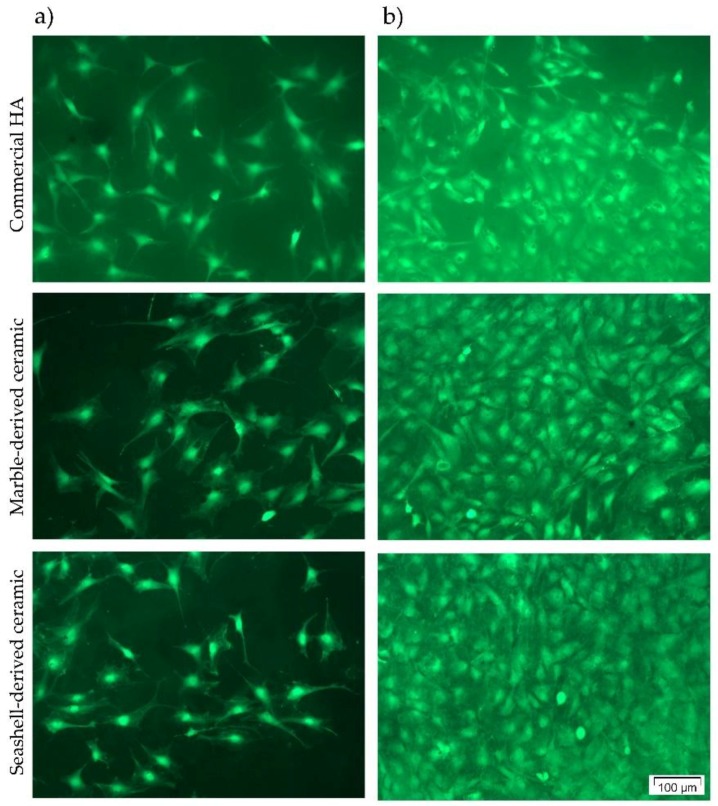
Fluorescence microscopy images of the MC3T3-E1 cells grown in contact with commercial HA and marble- and seashell-derived ceramics for 1 day (**a**) and 4 days (**b**). The cells were stained with a LIVE/DEAD Cell Viability/Cytotoxicity Assay Kit (live cells fluorescence green; no red fluorescent dead cells are present). Scale bar: 100 µm.

To finally assess the capacity of the developed ceramic materials to support pre-osteoblast viability and proliferation, the results of the LDH and MTT assays have been combined with the qualitative evaluation of MC3T3-E1 cells’ viability and densities by performing a LIVE/DEAD Cell Viability/Cytotoxicity assay. As shown in Figure 6, the pre-osteoblasts grown in contact with all three ceramics converted non-fluorescent calcein AM to green-fluorescent calcein, revealing a high percentage of viable cells. Thus, no red fluorescent dead cells and an increasing number of green fluorescent viable cells could be observed along the culture period. These findings are in agreement with the results of the LDH and MTT assays and, collectively, suggest that marble- and seashell-derived ceramics exhibited good cytocompatibility and promoted the proliferation of pre-osteoblast cells to a similar extent with the reference material. 

#### 3.2.2. MC3T3-E1 Cell Adhesion and Morphological Features

**Figure 7:**
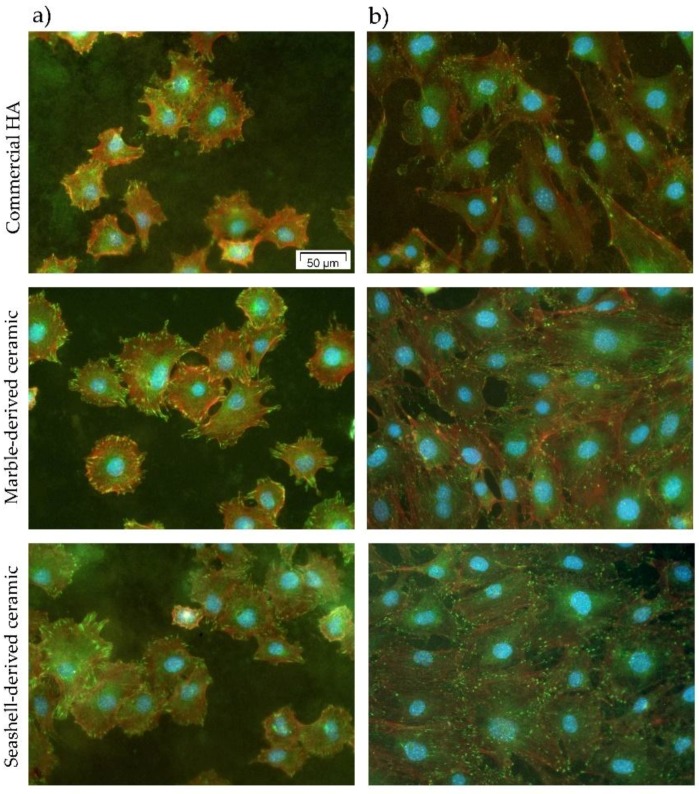
Merged fluorescence micrographs of actin cytoskeleton (red) and vinculin immunoreactive sites (green) in MC3T3-E1 pre-osteoblasts grown on commercial HA, marble- and seashell-derived ceramics for 3 h (**a**) and 24 h (**b**). The nuclei are labeled with DAPI (blue). Scale bar: 50 μm.

For an improved understanding of the cellular interactions with the analyzed calcium phosphate-based materials, the cell adhesion and morphology were microscopically investigated after double fluorescent staining of vinculin and actin filaments. Figure 7 and Figure S1 (Supplementary Material) show the morphological features of MC3T3-E1 pre-osteoblasts on these surfaces at 3 h and 24 h post-seeding. Three hours following cell seeding, all pre-osteoblasts were attached to the samples’ surfaces, and started to spread extending lamellipodia and filopodia in multiple directions (Figure 7a and Figure S1a). At the level of these cellular protrusions, discrete green-fluorescent vinculin signals can be seen on all analyzed substrates. Vinculin is an intracellular protein present in cadherin-mediated cell junctions and in focal adhesions playing a key role in initiating and establishing cell adhesion and cytoskeletal development [41]. It is worth mentioning that these immunoreactive signals are numerous on the marble- and seashell-derived ceramics suggesting the formation of focal adhesion contacts on these surfaces. Almost similar behavior was observed for commercial HA. As is well known, cell adhesion represents a cellular process accompanied by the rearrangement of cytoskeletal proteins, formation of tight focal adhesion contacts, activation of focal adhesion kinase (FAK) and induction of various intracellular signal transduction pathways that regulate cell survival, proliferation and differentiation [42,43]. Hence, we can conclude that the two newly synthesized bioceramics are as effective as an HA surface in promoting cell adhesion and establishing tight interactions with MC3T3-E1 pre-osteoblasts. At the 24 h time point (Figure 7b and Figure S1b), well-spread cell morphologies were displayed on all surfaces, but the pre-osteoblasts attached to HA surface exhibited more stretched and elongated shapes and more discrete punctiform vinculin signals than on the marble-derived and, especially, the seashell-derived ceramics (Figure S1b). Likewise, fluorescence images showed a circumferential localization of actin filaments near the cell membrane at 3 h after cell plating (Figure 7a and Figure S1a) and well-defined thin stress fibers in the cell body at 24 h post-seeding (Figure 7b and Figure S1b) on all studied surfaces suggesting that they promote maturation of the actin cytoskeleton.

#### 3.2.3. Pre-Osteoblast Differentiation Potential

The biological performance of the developed calcium phosphate-based materials was also evaluated by studying their potential to promote pre-osteoblast differentiation both in the absence (−OM) and presence (+OM) of the osteogenic medium containing β-GP and AA. The osteoconductive and osteoinductive effects of calcium phosphate ceramics are well documented [37,38,39,44] although the mechanisms through which cell differentiation is mediated are still incompletely known. In this context, the present manuscript sought to reveal the influence of the synthesized ceramics on the activity of intracellular ALP and collagen synthesis and deposition on the cell supporting materials.

**Figure 8:**
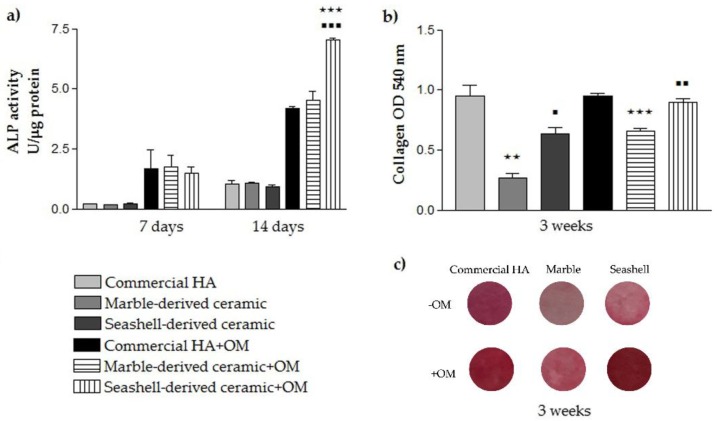
Results of the MC3T3-E1 cell differentiation assays showing: (**a**) the levels of intracellular ALP activity at 7 and 14 days post-seeding (*n* = 3, mean ± SD). ⋆⋆⋆ *p* < 0.001 for seashell-derived ceramic vs. commercial HA at 14 days post-seeding (+OM); ▪▪▪ *p* < 0.001 for seashell-derived ceramic vs. marble-derived ceramic at 14 days post-seeding (+OM); (**b**) Spectrophotometric quantification of the collagens deposited on the ceramic substrates (based on Sirius Red staining) (*n* = 3, mean ± SD). ⋆⋆ *p* < 0.01 for marble-derived ceramic vs. commercial HA (−OM); ⋆⋆⋆ *p* < 0.001 for marble-derived ceramic vs. commercial HA (+OM); ▪ *p* < 0.05 for seashell-derived ceramic vs. marble-derived ceramic (−OM); ▪▪ *p* < 0.01 for seashell-derived ceramic vs. marble-derived ceramic (+OM); (**c**) Digital images of the analyzed samples showing the staining of the collagenous matrix with Sirius Red.

As seen in Figure 8a, ALP activity of MC3T3-E1 cells grown on all analyzed ceramics continuously increased over the incubation period. After 7 days of culture, ALP activity was low whereas at 14 days post-seeding enhanced values were noticed for each cellular substrate in both experimental conditions. It is worthy to note that for all analyzed ceramic substrates, ALP activity was higher for pre-osteoblasts grown in osteogenic culture conditions versus standard conditions. Moreover, no significant differences occurred between samples except for the high enhancement of ALP activity in cells grown for 14 days under osteogenic conditions in contact with seashell-derived ceramic as compared to reference material and marble-derived ceramic (*p* < 0.001). ALP is considered an early marker of osteoblast differentiation involved in bone calcification. Specifically, ALP expression reaches a maximum level during the phase of matrix maturation, just before the onset of bone mineralization [45]. Therefore, based on the results obtained it can be concluded that marble- and seashell-ceramics are as effective as reference biomaterial in inducing matrix mineralization. However, a noticeable phenomenon was that, under osteogenic culture conditions, the ALP activity of MC3T3-E1 pre-osteoblasts was significantly higher (*p* < 0.001) on the seashell-derived ceramic than on the other analyzed calcium phosphate based-materials.

To further establish osteogenic commitment of MC3T3-E1 pre-osteoblasts, the collagenous matrix deposited on the analyzed materials was qualitatively and quantitatively measured by Sirius Red staining. Collagens (mainly type I collagen) are the major proteins which are related to bone extracellular matrix formation. They are synthesized and secreted during the initial period of proliferation and extracellular matrix biosynthesis. Annaz et al. [46] showed that the osteoblasts are intimately connected to calcium phosphates owing to the production of extracellular collagen firmly attached to their surfaces. Our results (Figure 8b,c) showed that the amount of collagen deposited on the seashell-derived ceramic after 3 weeks of culture was similar to that expressed on the reference material in the osteogenic culture conditions. However, in standard culture conditions, less collagen (*p* < 0.05) was quantified on the seashell-derived ceramic. It is worth mentioning that independent of culture conditions, marble-derived ceramic exhibited lower amounts of collagen in comparison to the reference material and the seashell-derived ceramics. Overall, all experiments performed in this study revealed that marble-derived ceramic elicited the less favorable pre-osteoblast response in terms of bone tissue integration whereas seashell-derived ceramic exhibited almost a similar pre-osteoblast response to commercial HA. Therefore, although both developed naturally derived calcium phosphate-based materials proved to be biocompatible, seashell-derived ceramic shows great promise for substituting commercial HA in the reconstructive orthopedic field. 

We assume that a surface characteristic responsible for this cellular behavior is wettability with the marble-derived ceramic displaying a water contact angle (CA) of 40° that denotes a more hydrophilic surface than seashell-derived ceramic (CA of 57°) and commercial HA (61°). It is a general trend that the cells exhibit better interaction with moderately hydrophilic substrates than hydrophobic or very hydrophilic ones [47]. Kim et al. [48] showed that the optimal wettability for cell adhesion that strongly influences subsequent cell behavior is a CA range of 50–60°. This can explain the similar and even better MC3T3-E1 pre-osteoblast response elicited by commercial HA and seashell-derived ceramic exhibiting CA values within this range.

## 4. Conclusions

In this work, the biological in vitro performance of recently developed naturally derived calcium phosphate-based materials, namely marble- and seashell-derived ceramics were comparatively assayed and related to commercial HA material. 

Results summarize on the one hand, the potential of the adapted Rathje method for naturally derived calcium phosphates synthesis, even from the use of marble precursors, and on the other hand, the importance of thermal treatment for modulated, enhanced morpho-compositional and surface features.

Further, our studies confirmed the cytocompatibility, osteoconductivity and osteoinductivity of the commercial HA material and revealed that the newly synthesized seashell-derived ceramic exhibited a slightly better MC3T3-E1 pre-osteoblast response.

It was demonstrated that there were no distinguishable differences in the survival/ proliferation rates and adhesion/morphological features of the cells attached to the two developed naturally derived calcium-phosphate-based materials. However, seashell-derived ceramic demonstrated a higher efficacy in inducing pre-osteoblast differentiation which exhibits the potential applications these may have in the reconstructive orthopaedic field. 

## Figures and Tables

**Table 1:** Chemical composition of naturally derived calcium phosphate based-materials: (A) post Synthesis and (B) post Thermal Treatment and compressive strength of sintered pellets.

Sample Type	O (at. %)		Mg (at. %)		P (at. %)		Ca (at. %)		Ca/P ratio		Compressive Strength (N/mm^2^)
	(A)	(B)	(A)	(B)	(A)	(B)	(A)	(B)	(A)	(B)	
Marble	55.10	54.04	0.58	1.28	17.71	16.65	26.61	26.64	1.50	1.60	2.37
Seashell	60.76	55.99	-	-	15.51	15.81	23.73	25.61	1.53	1.62	4.53

## Supplementary Materials

The following are available online at http://www.mdpi.com/1996-1944/11/7/1097/s1: Figure S1: Fluorescent images of MC3T3-E1 pre-osteoblasts grown on commercial HA, marble- and seashell-derived ceramics for 3 h (a) and 24 h (b) respectively. Red fluorescence: actin cytoskeleton; Green fluorescence: vinculin signals. Scale bar: 50 µm.
