# Supplementary material for: Osteoblast Cell Response to Naturally Derived Calcium Phosphate-Based Materials
Source: Materials (Basel). 2018 Jun 27;11(7):1097. doi: 10.3390/ma11071097 (PMC6073128; doi:10.3390/ma11071097)
Supplement: Supplementary file 1 [file materials-11-01097-s001.pdf]

materials

Supplementary Material

Osteoblast cell response to naturally derived calcium phosphate-based materials

Valentina Mitran [1], Raluca Ion [1], Florin Miculescu [2,*], Madalina Georgiana Necula [1], Aura-Catalina Mocanu [2,3], George Stan [4], Iulian Antoniac [2], Anisoara Cimpean [1,*]

1 University of Bucharest, Department of Biochemistry and Molecular Biology, 91-95 Spl. Independentei, 050095 Bucharest, Romania; valentinamitran@yahoo.com (V.M); rciubar@yahoo.com (R.I); necula.madalina92@gmail.com (M.G.N.); anisoara.cimpean@bio.unibuc.ro (A.C.)
2 University Politehnica of Bucharest, Department of Metallic Materials Science, Physical Metallurgy, 313 Splaiul Independentei, 060042, J Building, District 6, Bucharest, Romania; f_miculescu@yahoo.com (F.M.); mcn_aura@hotmail.com (A.C.M.); antoniac.iulian@gmail.com (I.A.)
3 S.C. Nuclear NDT Research & Services S.R.L, Department of Research, Development and Innovation, 104 Berceni Str., Central Laboratory Building, District 4, Bucharest, Romania; aura.mocanu@nuclearndt.ro (A.C.M.)
4 National Institute of Materials Physics, Laboratory of Multifunctional Materials and Structures, Atomistilor Str., No. 405A PO Box MG 7, 077125, Măgurele-Bucharest, Romania; george_stan1@yahoo.com (G.S)

* Correspondence: anisoara.cimpean@bio.unibuc.ro; Tel.: +40-21-3181575/106; f_miculescu@yahoo.com; Tel.: +40-21-3169563

Received: 6 June 2018; Accepted: 25 June 2018; Published: 27 June 2018

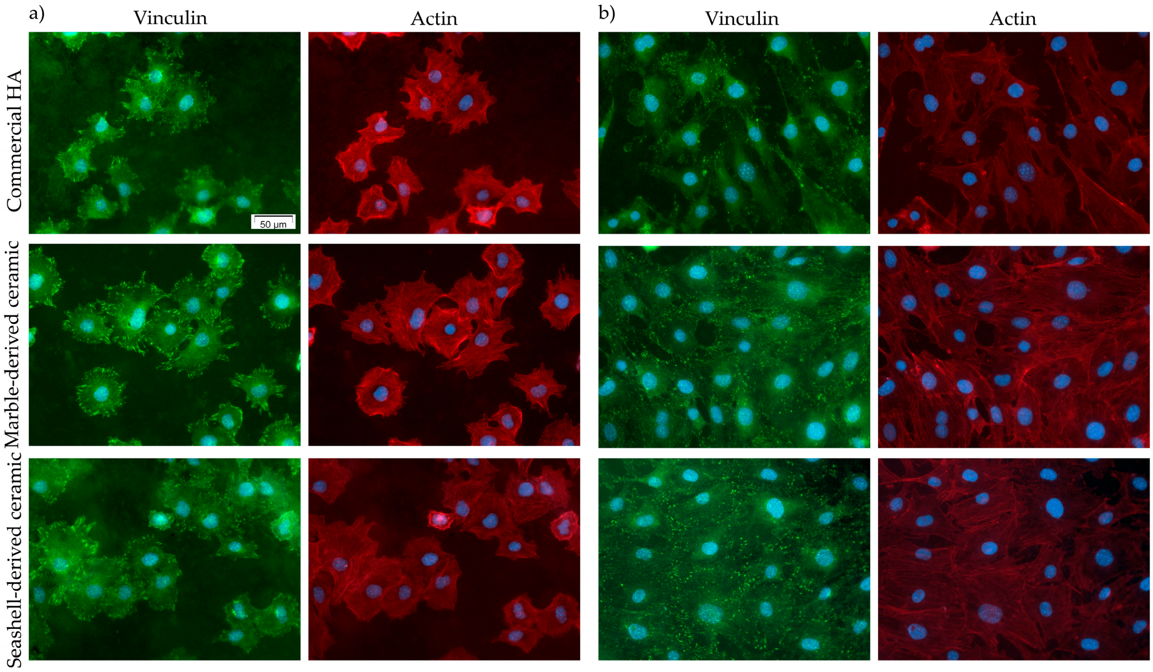

Figure S1. Fluorescent images of MC3T3-E1 pre-osteoblasts grown on commercial HA, marble- and seashell-derived ceramics for 3 h (a) and 24 h (b) respectively. Red fluorescence: actin cytoskeleton; Green fluorescence: vinculin signals. Scale bar: 50 µm.
